# Management of Liposarcoma With Posterior Intercostal Artery Propeller Flap Reconstruction: A Case Report

**DOI:** 10.7759/cureus.62346

**Published:** 2024-06-13

**Authors:** Krushank Nayak, Firoz Borle, Chava Aravind Kumar

**Affiliations:** 1 General Surgery, Jawaharlal Nehru Medical College, Datta Meghe Institute of Higher Education and Research, Wardha, IND

**Keywords:** reconstructive surgery, oncologic surgery, flap surgery, surgical management, soft tissue sarcoma, case report, propeller flap reconstruction, posterior intercostal artery, right flank, large liposarcoma

## Abstract

This case shows the administration of a 57-year-old male with liposarcoma within the right flank region. Surgical treatment of the case included wide local excision (WLE), taken after reconstruction utilizing a posterior intercostal artery propeller flap. Postoperative care included regular checking for signs of repeat. Comparison with similar cases highlights the changeability in clinical introduction and surgical approaches for liposarcomas. This case emphasizes the significance of convenient diagnosis, fastidious surgical procedures, and successful reconstruction in overseeing liposarcomas. This case report points to highlights the clinical administration, surgical intercession, and postoperative care included in treating a giant liposarcoma and compares this case with similar instances to emphasize the challenges and procedures in treating liposarcomas.

## Introduction

A benign, slowly growing lump of fatty tissue that is circular or oval and ordinarily feels smooth to the touch and moves when touched is called a lipoma [1]. The regions that are most regularly found are the back, trunk, arms, shoulders, and neck [1]. One sort of cancer that starts within the body's connective tissues-muscles, bones, cartilage, and fat is called a sarcoma [2]. Unlike lipomas, benign tumours are composed of fat cells, and sarcomas are dangerous tumours that can attack encompassing tissues and spread to other body parts [3]. Sarcomas can be classified into soft tissue and bone sarcomas [1,4]. Bone sarcomas, such as chondrosarcoma, Ewing's sarcoma, and osteosarcoma, primarily affect the long bones, while soft tissue sarcomas, including liposarcoma, leiomyosarcoma, and angiosarcoma, can develop from various soft tissues throughout the body [4,5]. Wide local excision (WLE) may be a common surgical treatment for soft tissue sarcomas, counting liposarcoma [1]. This treatment disposes of minuscule cancer cells from the tumour and the encompassing healthy tissue, minimizing the chance of repeat [1,2]. The posterior intercostal artery propeller flap (PIAPF) is a flexible surgical procedure utilized for reconstructive methods, especially within the thoracic and back regions, which receives blood from the posterior intercostal supply channels, which drives the PIAPF into the imperfection range [6].

## Case presentation

**Figure 1:**
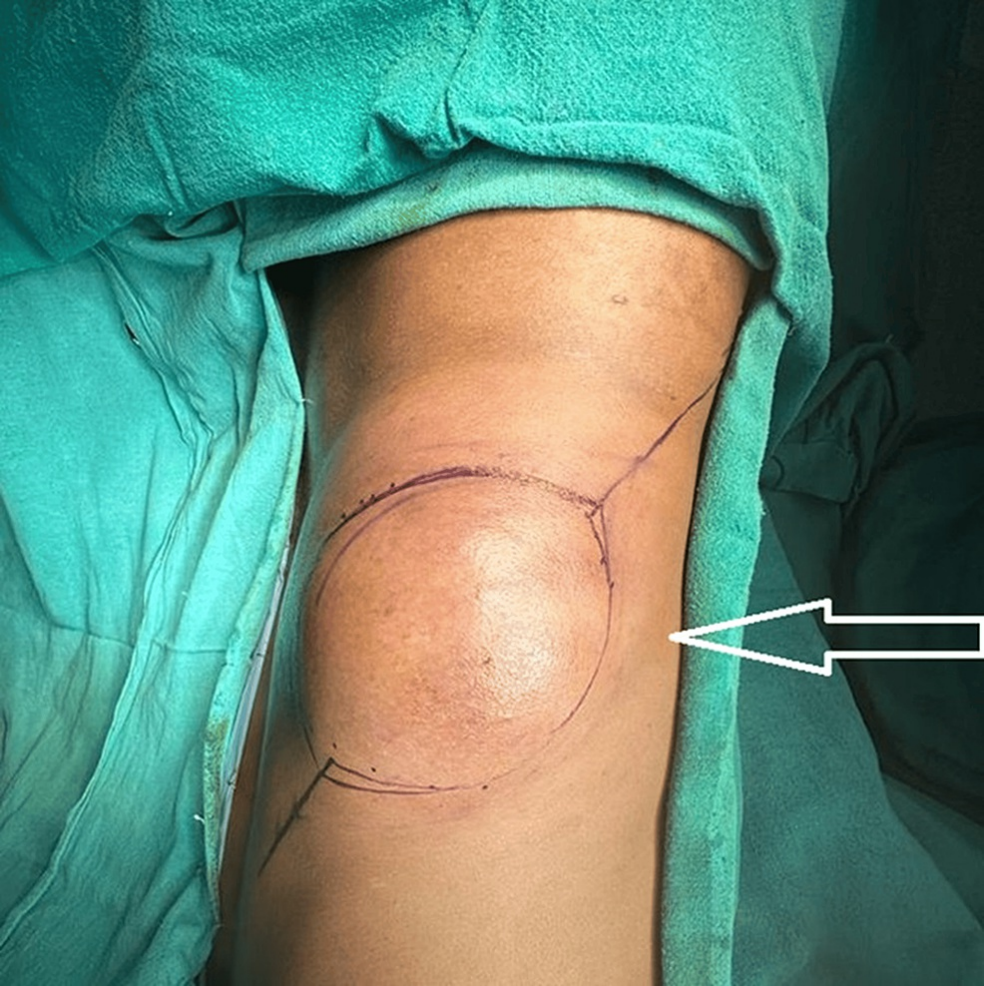
Swelling of the right flank region The white arrow represents a swelling on the patient's right flank.

A 57-year-old male presented to the OPD of Acharya Vinoba Bhave Rural Hospital (AVBRH) with a chief complaint of swelling on the right side of his flank. The patient reported that the swelling had been present for the past two years, and there was no pain associated with the swelling. It had an insidious onset and had progressively increased in size over time. The swelling was approximately 15x08 cm upon examination (Figure 1).

**Figure 2:**
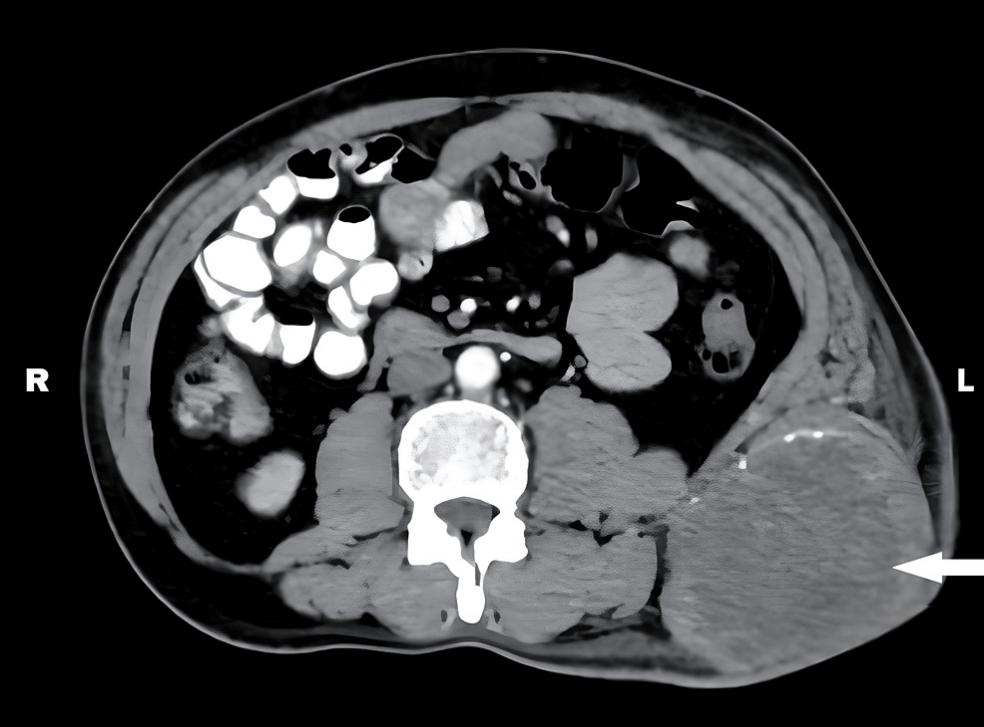
CECT scan of the abdomen and pelvis A contrast-enhanced CT (CECT) scan of the abdomen and pelvis revealed a lesion in the anterior abdominal wall, as shown with the white arrow.

A contrast-enhanced CT (CECT) scan of the abdomen and pelvis revealed a lesion in the anterior abdominal wall (Figure 2).

**Figure 3:**
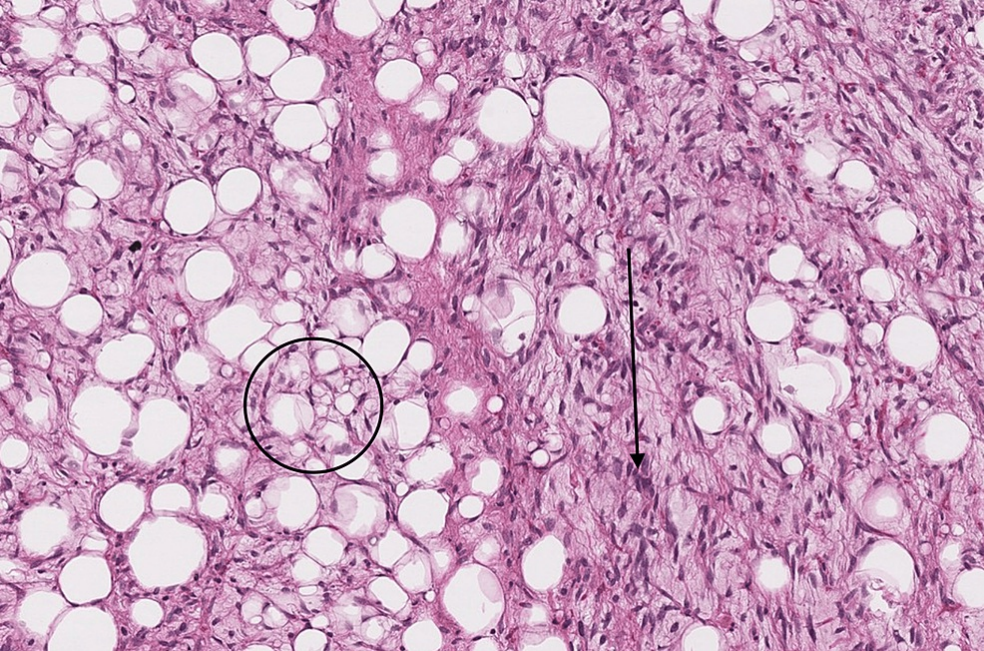
Histopathological image of liposarcoma Hematoxylin and eosin (H&E), 40x; section from the mass in the flank region shows immature adipocytes (black circle) separated by connective tissue that has a loose myxoid (black arrow)

Notably, there was no lesion extension into the muscular or intra-abdominal regions. To further investigate the nature of the lesion, a biopsy was taken. A histopathological examination of the biopsy confirmed the diagnosis of liposarcoma (Figure 3).

**Figure 4:**
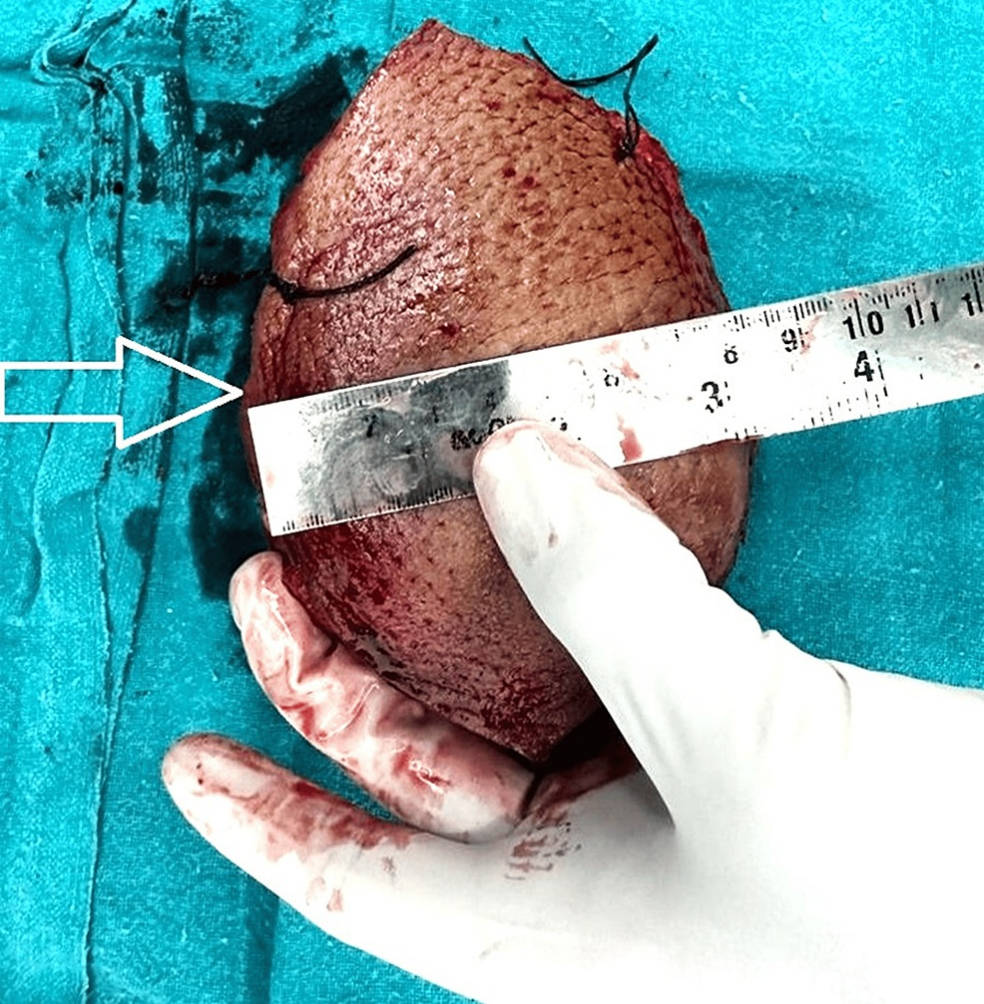
The specimen of excised liposarcoma The specimen of excised liposarcoma is depicted with a white arrow

Given the diagnosis, the primary treatment plan involved the surgical excision of the tumour. It was decided to perform a WLE with a 2 cm margin of normal tissue around the tumour to ensure complete removal. Post-excision defect reconstruction was planned through a PIAPF. The patient was brought to the operating room and placed under general anaesthesia. The surgical site was prepped and draped in a sterile manner. An elliptical incision around the tumour ensured a 2 cm margin of normal tissue. The tumour was carefully dissected and excised completely. Haemostasis was meticulously achieved using electrocautery. The excised tissue was sent for histopathological analysis to confirm clear margins. The specimen of excised liposarcoma is shown in Figure 4.

**Figure 5:**
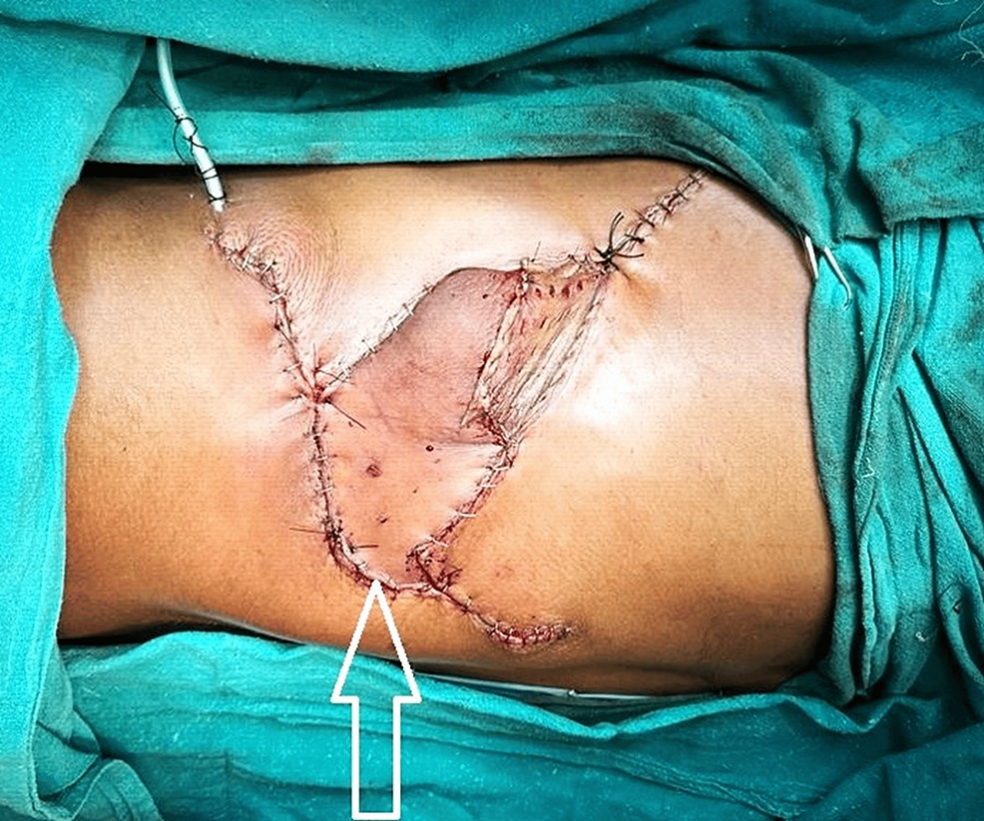
Posterior intercostal artery propeller flap A posterior intercostal artery propeller flap utilized is depicted with a white arrow

For the reconstruction, a PIAPF was chosen because of its reliable blood supply and suitability for covering large defects. The flap was carefully planned, with markings made on the donor site to delineate the dimensions and arc of rotation. The posterior intercostal supply route and its perforators were recognized and protected amid the flap height. The flap was elevated while guaranteeing the vascular pedicle was intaglio. The following step included pivoting the flap 180 degrees to cover the imperfection made by the tumour extraction. This turn posed a critical challenge because of maintaining a satisfactory blood supply and a strategic distance from kinking or torsion of the vascular pedicle. At that point, the flap was carefully situated and sutured at the deformity location, guaranteeing no pressure on the pedicle. Depending on the imperfection size, the benefactor site was closed essentially or with a skin graft, and drains were put as required to avoid fluid accumulation. The surgical location was dressed in sterile dressings. A PIAPF is utilized (Figure 5).

Postoperatively, the patient was closely monitored for signs of flap viability, including observations of colour, temperature, and capillary refill. Regular wound checks and vital signs monitoring were conducted. Pain management was provided using analgesics, and prophylactic antibiotics were administered to prevent infection. The patient was scheduled for regular follow-up visits for two years to monitor healing and detect any signs of recurrence or complications. The timeline of events in the case is depicted in Table 1.

**Table 1:** The timeline of events in the case Table created by Krushank Nayak

Time	Event
Two Years Ago	Swelling first noted on the right side of the flank region
Day 1	Presentation to OPD with a 15x08 cm swelling
Day 2	Contrast-enhanced CT (CECT) scan of the abdomen and pelvis performed
Day 3	Biopsy of the lesion taken for histopathological examination
Day 10	Diagnosis of liposarcoma confirmed via histopathological examination
Day 20	Surgical excision planned, and patient scheduled for surgery
Day 25	Surgical excision performed under general anaesthesia
	Tumour excised with a 2 cm margin of normal tissue
	Posterior intercostal artery propeller flap used for reconstruction
	Haemostasis achieved, excised tissue sent for histopathological analysis
	Flap elevated, rotated 180 degrees, positioned, and sutured into place
	Donor site closed, and drains placed as needed
Postoperative Day 1	Patient monitored for signs of flap viability, colour, temperature, and capillary refill
Postoperative Day 2	Regular wound checks, vital signs monitoring, pain management, and prophylactic antibiotics administered
Postoperative Day 7	First follow-up visit, wound check, and assessment of healing
Monthly for 2 Years	Regular follow-up visits to monitor for healing, recurrence, or complications

## Discussion

In the case of a 52-year-old male patient reported by Ataya et al., a well-defined low-grade liposarcoma in the posterior mediastinum was observed, which spread to the hilum and upper lobe of the right lung [7]. He was brought up with progressive exertional dyspnea, dry cough, tiredness, and unexpected weight loss for three months. Physical examination revealed diminished breath sounds and dullness to percussion in the upper right lung. A chest X-ray and contrast-enhanced CT scan confirmed the presence of a large heterogeneous mass generating extrinsic compression of the right lung, heart, large vessels, and oesophagus [7]. Histopathological examination revealed a mass with chondroid metaplasia and limited ossification, with immunohistochemical positivity for MDM2 and CDK4, confirming the diagnosis of a well-differentiated liposarcoma [7].

Conversely, in our case, a 57-year-old male patient appeared with a bulge on the right side of his flank region, which had been developing over two years. The mass measured approximately 15x08 cm. A contrast-enhanced CT scan of the abdomen and pelvis revealed a lesion in the anterior abdominal wall. Both patients required significant surgical intervention. The 52-year-old patient underwent a right thoracotomy with a radical excision of the mediastinal lesion [7]. The procedure kept the trachea and oesophagus intact while the bulging azygos vein was ligated and excised. The lobulated mass was successfully resected, and postoperative recovery was rapid, the chest tube was withdrawn on the fourth day, and a clear follow-up chest X-ray was done [7].

Our 57-year-old patient, after WLE, required reconstructive surgery employing a propeller flap. In an outline, whereas both cases include liposarcomas requiring surgical removal, they contrast essentially in the tumour area, clinical introduction, and a few perspectives of surgical and postoperative administration [7]. The mediastinal liposarcoma was related to serious respiratory side effects and noteworthy structural compression. However, the abdominal wall liposarcoma displayed as a localized swelling without systemic side effects, highlighting the shifted clinical challenges posed by liposarcomas in several anatomical settings.

A case of the 55-year-old female patient reported by Deng et al. with a giant liposarcoma on the back of her left thigh presents both similarities and differences compared to our case with a liposarcoma on the right side of his flank [8]. The 55-year-old female patient's tumour had been shown for six years but had strikingly expanded in estimate over the final year, prompting her to look for restorative consideration [8]. Essentially, the male patient's tumour had been developing over a period of two years. Both tumours were expansive, with the female patient measuring around 24x15x15 cm and our male patient measuring 15x08 cm [8]. Diagnostic imaging played a significant part in both cases. For the 55-year-old female patient, CT and MRI scans uncovered a huge space-occupying lesion with generally fat density, steady with a liposarcoma [8]. The male patient was affirmed through a contrast-enhanced CT (CECT) scan of the abdomen and pelvis, which recognized the lesion within the anterior abdominal wall. Within the female patient, the tumour displayed characteristics of well-differentiated liposarcoma, as evidenced by immunohistochemistry appearing CDK4 partial positivity and changed adipocyte sizes with a few huge nuclei and profoundly stained stromal cells [8].

Surgical intervention was the chosen treatment for both patients. The female patient underwent resection of the tumour under lumbar anaesthesia. The tumour was cystic, irregularly shaped, and encapsulated during the operation, with extensive adhesion to surrounding tissues, including the femoral nerve and blood vessels [8]. The tumour, along with some muscle tissue, was completely removed. The male patient had a WLE of the tumour. A PIAPF was used during the operation. Despite variations in tumour site and surgical strategy, both cases demonstrate the importance of meticulous surgical planning and intervention in treating liposarcoma [8].

The case of the 80-year-old man reported by Itagaki et al. and our case of a 57-year-old male with a swelling in the right flank share similarities and differences, particularly in their presentation, diagnostic findings, and treatment approach [9]. Both cases involve liposarcomas, although they are located in different anatomical regions. The 80-year-old patient displayed a mass on the right side of his chest and related flank pain, alongside gastrointestinal side effects that had gone before the primary visit [9]. His therapeutic history included critical comorbidities such as hypertension, dyslipidemia, combined pulmonary fibrosis and emphysema, and a past cerebral infarction. An overwhelming ex-smoker with a 45-pack-year history, his chest CT scan uncovered a huge, low-density, heterogeneous tumour disintegrating the eighth rib and containing marginally dense fluid and calcifications, proposing a complex injury [9]. Despite a needle biopsy demonstrating conceivable sarcoma, a conclusive pathological diagnosis was at first slippery.

In contrast, our case of a 57-year-old male had a longstanding swelling over the right flank for two years, significantly larger at approximately 15x08 cm. A contrast-enhanced CT (CECT) scan confirmed the lesion in the anterior abdominal wall. Histopathological examination of the biopsy confirmed the diagnosis of liposarcoma, specifically a dedifferentiated subtype similar to the 80-year-old patient's tumour [9]. The 80-year-old patient underwent total surgical excision of the chest wall tumour repaired with a polytetrafluoroethylene mesh [9]. His resected tumour showed biomorphic features with necrotic foci and high murine double minute 2 (MDM2) and CDK4 expression, confirming dedifferentiated liposarcoma (DDLPS). Immunohistochemical examination indicated T-cell infiltration and elevated programmed death-ligand 1 (PD-L1), indicating an immune response within the tumour microenvironment [9]. Both cases emphasize the importance of thorough surgical excision and appropriate reconstruction to manage large, complex tumours such as liposarcomas [9]. Despite the spontaneous regression observed in the older patient's tumour, surgery remained necessary because of the potential for malignancy.

A case of an 87-year-old Greek man reported by Paraskeva et al. with a giant swelling on his back present for at least 10 years [10]. This tumour was asymptomatic, leading to delayed surgical intervention. A chest CT scan revealed a giant liposarcoma measuring 18 × 14 × 6 cm, which was removed under regional anaesthesia [10]. The pathology report uncovered different neoplastic components, counting non-differentiated liposarcoma, well-differentiated liposarcoma, malignant fibrous histiocytoma, osteosarcoma, chondrosarcoma, and pericellular pattern regions [10]. The perioperative period was uneventful, and the patient was released three days postoperatively. In comparison, our case includes a 57-year-old male with swelling over the right flank region, shown for two years. This swelling measured approximately 15 × 10 cm, and a contrast-enhanced CT (CECT) scan revealed a lesion in the anterior abdominal wall. Histopathological examination confirmed the diagnosis of liposarcoma. After WLE, a PIAPF was utilized for reconstruction.

Both cases involve large liposarcomas requiring surgical excision. The 87-year-old patient's tumour had an essentially longer history and a bigger size compared to our patient's tumour, which was identified and treated in two years [10]. The 87-year-old's tumour exhibited different histological types, demonstrating a more complex neoplastic profile, while the 57-year-old's tumour was exclusively distinguished as a liposarcoma [10]. Moreover, the older patient's surgery was performed under regional anaesthesia because of his age, whereas our patient's surgery included general anaesthesia [10].

## Conclusions

This case describes the correct treatment of a 57-year-old male with liposarcoma on the right flank, treated with a WLE. The PIAPF empowered fitting deformation. A multidisciplinary approach and solid long-term observation are basic for progressing treatment results and surveying prognosis, underlining the potential for future progress in persistent care and quality of life.
